# Alopecia Universalis following Alemtuzumab Treatment in Multiple Sclerosis: A Barely Recognized Manifestation of Secondary Autoimmunity—Report of a Case and Review of the Literature

**DOI:** 10.3389/fneur.2017.00569

**Published:** 2017-10-30

**Authors:** Julian Zimmermann, Timo Buhl, Marcus Müller

**Affiliations:** ^1^Department of Neurology, Universitätsklinikum Bonn, Bonn, Germany; ^2^Department of Dermatology, Venereology and Allergology, University Medical Center Göttingen, Göttingen, Germany

**Keywords:** alemtuzumab, multiple sclerosis, alopecia universalis, alopecia areata, alopecia totalis, treatment safety, adverse events, autoimmunity

## Abstract

Secondary autoimmunity is the most frequent adverse event occurring in almost every other alemtuzumab-treated multiple sclerosis patient. We report a case of a patient with relapsing-remitting multiple sclerosis who reported smooth, circular areas of complete hair loss on both thighs 6 months after the second treatment cycle with alemtuzumab. The patient was diagnosed as having alopecia areata (AA). Within 3 months, AA progressed to complete loss of all body hair (alopecia universalis). Current literature rarely connects alemtuzumab with the onset of alopecia of autoimmune origin. Here, we report a little-noticed autoimmune disease affecting the skin, very likely being associated with alemtuzumab. We emphasize the necessity of careful clinical surveillance of alemtuzumab-treated patients for yet undescribed autoimmune diseases.

## Background

Alemtuzumab is a monoclonal antibody targeting the CD52 surface antigen, thereby depleting all mature lymphocytes. It is a highly effective agent for the treatment of relapsing-remitting multiple sclerosis (RRMS). Alemtuzumab has proven superior efficacy over interferon beta 1a (1–3). The most significant adverse event relies to autoimmunity secondary to alemtuzumab application. According to several published long-term treatment data, around 47% of the patients develop another autoimmune disorder apart from MS. Most commonly, thyroid disorders (35–41%) and immune thombocytopenia occur (3–3.5%) (4–7). In addition, autoimmune renal diseases are observed at a lower frequency but may require renal transplantation.

Alopecia is a common adverse event in several MS treatment options such as teriflunomide or mitoxantrone. The pathobiology of this chemotherapy induced hair-loss is well known (8). Though alopecia is also described in alemtuzumab-treated patients the mechanisms of hair loss have not been discussed in current literature. Here we provide evidence, that alopecia is an additional, but barely recognized secondary autoimmune disease after alemtuzumab application.

## Case Report

**Figure 1:**
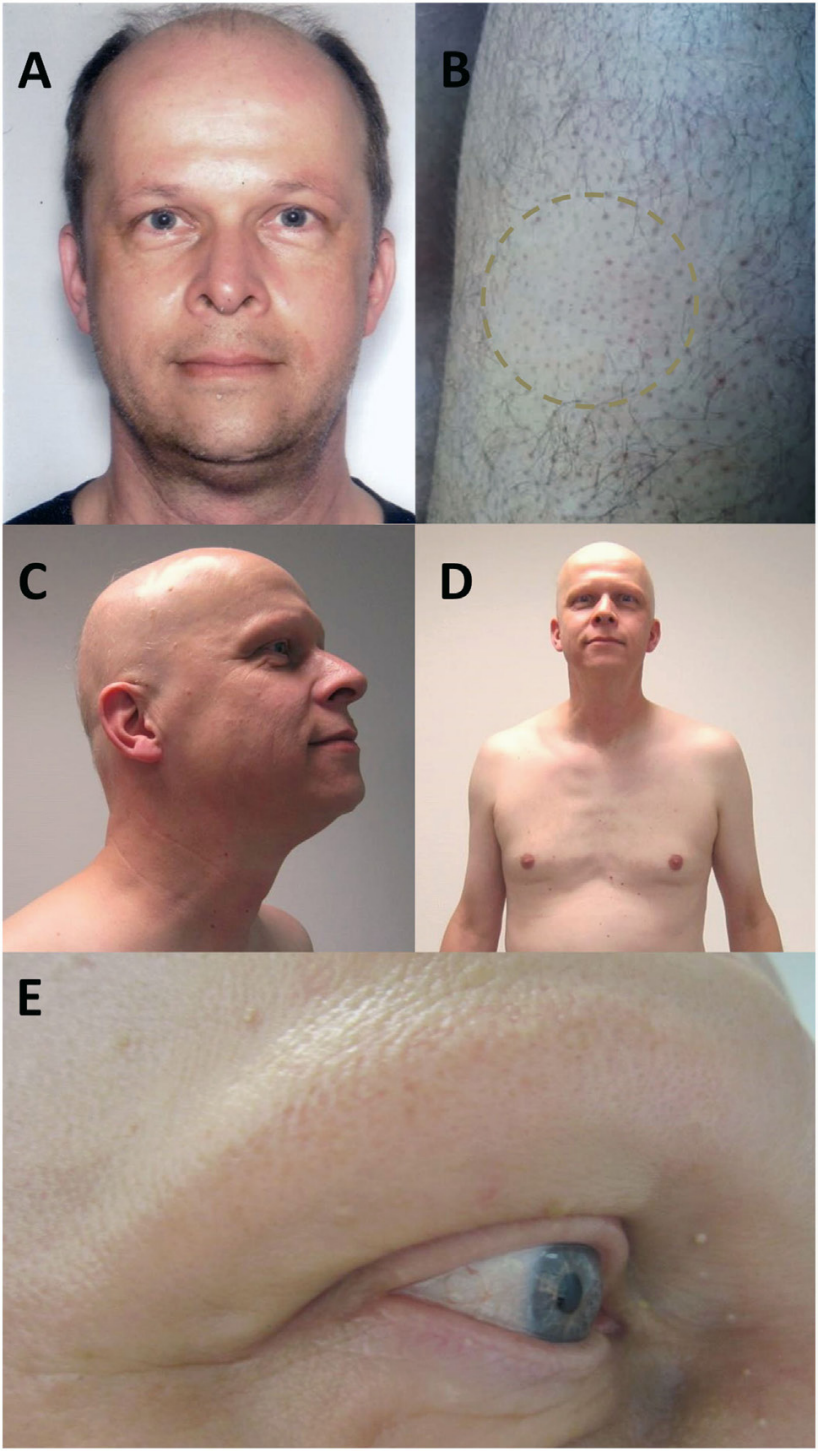
Photograph of the patient before onset of alopecia, after first alemtuzumab cycle **(A)**. Six months after second alemtuzumab cycle, the patient presented with smooth, circular areas of complete hair loss at both thighs **(B)**. Three months later, the alopecia progressed to a complete loss of all scalp hair with a few remaining thin and pigmented residual hairs **(C)** and a patchy body hair loss including chest **(D)**, axillae, and pubic region. Eyebrows and eyelashes were extensively involved **(E)**.

A 49-year-old Caucasian man received two cycles of alemtuzumab for his RRMS. Prior to alemtuzumab treatment, the patient received interferon-beta-Ia i.m., interferon-beta-Ib s.c., and 11 cycles of mitoxantrone with a cumulative dose of 71 mg/m^2^ without relevant hair loss (Figure 1A). Medical history excluded any other autoimmune disease besides RRMS. Since initiation of alemtuzumab treatment, MS-disease activity remained stable with an expanded disability status scale of 4.0. Six months after the second cycle, the patient reported newly emerged smooth, circular areas of complete hair loss at both thighs (Figure 1B). He denied having had similar symptoms before. Consultation of a dermatologist resulted in the diagnosis alopecia areata (AA) based on the classical clinical presentation. Three months later, the patient complained of progression of alopecia to a patchy body hair loss and complete loss of all scalp hair including eyebrows and eyelashes (Figures 1C–E). During dermatological reassessment, the patient presented with non-scarring alopecia including scalp, chest, both thighs, axillae, and pubic region. A few thin and pigmented residual hairs, around 3 cm long, were remaining on the scalp. Alopecia totalis was diagnosed and steroid treatment discussed. However, the patient abstained from further therapies. All laboratory tests including thyroid hormones and thyroid antibodies, as well as total blood count, revealed no abnormalities throughout the disease course. Regrowth of hair did not occur for a follow-up period of 6 months. The patient provided written informed consent for publication of his case history and images.

## Literature Search

Regarding to the risk of alopecia during alemtuzumab treatment, both pivotal trials CARE-MS I and CARE-MS II did not report alopecia as a common adverse event (1, 2). Nevertheless, alemtuzumab’s EMA label^1^ refers to alopecia as a common side effect in 1–10 people. FDA’s label^2^ does not mention alopecia as relevant adverse event. However, few reports account alopecia as an autoimmune event caused by alemtuzumab infusion. A recent publication on the 4–5-year long follow-up of 61 highly active MS patients reports one patient with “total alopecia” after alemtuzumab (9). Another recent observational study with 100 patients followed for 6.2 years refers to two patients as having alopecia as an autoimmune disorder after receiving alemtuzumab (5). A 41-year-old women with secondary autoimmune thyroiditis has been described as having “patchy alopecia” resembling initial pattern of the patient described here 34 month after initiation of alemtuzumab treatment (10). Taken together, in the literature we identified a second patient with alopecia universalis or alopecia totalis and three more with autoimmune-related alopecia after alemtuzumab therapy. Reviews focusing on secondary autoimmunity as well as alemtuzumab’s official labels do not recognize alemtuzumab-induced alopecia as of autoimmune origin. Furthermore, a PubMed search for “alemtuzumab” and “alopecia” or “hair loss” returns no results for the indication of multiple sclerosis.

## Discussion

Alopecia is a well-known adverse event of several MS therapies. Regarding teriflunomide treatment, a pooled safety and tolerability analysis from four placebo-controlled studies and extension studies with a cumulative exposure to teriflunomide >6,800 patient-years reported a dose-dependent hair thinning in 10.0–13.9% of all patients (11). Hair thinning occurred primarily during the first 6 months of teriflunomide treatment, no complete hair loss was reported, and most cases resolved while treatment was continued. Mitoxantrone treatment resulted in a 4.65-times higher risk of developing alopecia compared to placebo-treated participants. Altogether, 45.5% of all mitoxantrone-treated patients develop alopecia (12). Cyclophosphamide-treated patients have been described with a mild, reversible alopecia as the most frequent side effect observed in up to 48% of patients (13). Chemotherapy induced hair loss generally occurs early after treatment and is frequently reversible within 6 weeks after cessation (8).

Alopecia areata is an autoimmune disease resulting from T-cell mediated damage of the hair follicle (14). It is widely accepted that CD8+NKG2D+ T-cells infiltrate the hair follicle bulb leading to hair loss [reviewed in Ref. (15)]. Transfer of pathogenic T-cells, but neither B-cells nor sera, can cause the disease in human xenograft models (16). However, B-cell-mediated autoimmunity of the thyroid gland is clearly associated with AA (17–20). Especially, severe forms of AA, like alopecia totalis (loss of all scalp hair including eyebrows and eyelashes) and alopecia universalis (loss of all scalp and body hair) are strongly linked with thyroid autoimmunity ranging from 25 to 40% of all cases (17, 20). Other autoimmune diseases associated with AA are psoriasis (3.7%), vitiligo (1.4%), diabetes mellitus (1.4%), and rheumatoid arthritis (1.4%) (20). Though AA and MS share several genetic risk loci such as *CTLA4, IL-2/IL-21, IL-2RA*, association between these two autoimmune diseases is week (20–22).

The incidence of AA is high with 0.2/1,000 patients/year (23, 24). AA is more prevalent in younger patients suffering in 82.6–88% of all cases from their first AA onset before the age of 40 (25). Severe forms of AA like alopecia universalis and alopecia totalis occur in 7.2% of all cases, especially in the first two decades (26). Regarding clinical studies, all reported cases of alopecia totalis and universalis occurred before the age of 30.

Despite the high incidence rate of spontaneous AA, a plethora of facts argue for a causal relationship between AA and alemtuzumab in this reported case: (1) the autoimmune pathophysiology of AA, (2) the untypical clinical presentation with onset of alopecia universalis at the age of 49, (3) the identification of other cases of AA and especially alopecia universalis after alemtuzumab treatment in the literature, (4) the typical time course of secondary autoimmunity beginning from 18 months after the first infusion, and (5) the strong association between AA and thyroid autoimmunity on one hand, and the association of alemtuzumab treatment and secondary thyroid autoimmunity on the other hand. Regarding to the WHO-UMC^3^ system for standardized case causality assessment, the reported adverse reaction has to be graded as “probable/likely,” the second highest causality category.

## Concluding Remarks

Alemtuzumab treated MS patients are advised to examine themselves for signs of thrombocytopenia. Furthermore, they undergo a strict safety program for the detection of secondary autoimmunity. Taking into account the autoimmune origin of AA, alemtuzumab treated patients should be informed about the risk of developing alopecia. Besides screening for petechial bleeding, patients should be advised to examine themselves for possible hair loss to warrant early diagnosis and prompt topical or systemic steroid treatment. We propose recently developing AA as a red flag in alemtuzumab treated patients to screen for newly developing anti-thyroid antibodies and further signs of secondary autoimmunity.

## Author Contributions

JZ, TB, and MM contributed to design, analysis, and interpretation of the work. All authors drafted the manuscript, gave their final approval for publication, and agreed to be accountable for all aspects of the work.

## Conflict of Interest Statement

The authors declare that the research was conducted in the absence of any commercial or financial relationships that could be construed as a potential conflict of interest.

## Footnotes

^1^www.ema.europa.eu/docs/en_GB/document_library/EPAR_-_Product_Information/human/003718/WC500150521.pdf.

^2^www.accessdata.fda.gov/drugsatfda_docs/label/2014/103948s5139lbl.pdf.

^3^www.who-umc.org.

## References

[1] Cohen JA Coles AJ Arnold DL Confavreux C Fox EJ Hartung HP Alemtuzumab versus interferon beta 1a as first-line treatment for patients with relapsing-remitting multiple sclerosis: a randomised controlled phase 3 trial. Lancet (2012) 380:1819–28. 10.1016/S0140-6736(12)61769-3 23122652

[2] Coles AJ Twyman CL Arnold DL Cohen JA Confavreux C Fox EJ Alemtuzumab for patients with relapsing multiple sclerosis after disease-modifying therapy: a randomised controlled phase 3 trial. Lancet (2012) 380:1829–39. 10.1016/S0140-6736(12)61768-1 23122650

[3] CAMMS223 Trial Investigators Coles AJ Compston DA Selmaj KW Lake SL Moran S Alemtuzumab vs. interferon beta-1a in early multiple sclerosis. N Engl J Med (2008) 359:1786–801. 10.1056/NEJMoa0802670 18946064

[4] Tuohy O Costelloe L Hill-Cawthorne G Bjornson I Harding K Robertson N Alemtuzumab treatment of multiple sclerosis: long-term safety and efficacy. J Neurol Neurosurg Psychiatry (2015) 86:208–15. 10.1136/jnnp-2014-307721 24849515

[5] Willis MD Harding KE Pickersgill TP Wardle M Pearson OR Scolding NJ Alemtuzumab for multiple sclerosis: long term follow-up in a multi-centre cohort. Mult Scler (2016) 22:1215–23. 10.1177/1352458515614092 26514979

[6] Ruck T Bittner S Wiendl H Meuth SG. Alemtuzumab in multiple sclerosis: mechanism of action and beyond. Int J Mol Sci (2015) 16:16414–39. 10.3390/ijms160716414 26204829 PMC4519957

[7] Coles AJ Cox A Le Page E Jones J Trip SA Deans J The window of therapeutic opportunity in multiple sclerosis: evidence from monoclonal antibody therapy. J Neurol (2006) 253:98–108. 10.1007/s00415-005-0934-5 16044212

[8] Paus R Haslam IS Sharov AA Botchkarev VA. Pathobiology of chemotherapy-induced hair loss. Lancet Oncol (2013) 14:e50–9. 10.1016/S1470-2045(12)70553-3 23369683

[9] Ghodasara RS Smith SRS Mosley M Morgan R Bower M Kagan L Serious Adverse Events (SAE), Autoimmunity (AI), and Infections… by Dr. Samuel F. Hunter. (2017). Available from: http://onlinelibrary.ectrims-congress.eu/ectrims/2016/32nd/145865/samuel.f.hunter.serious.adverse.events.28sae29.autoimmunity.28ai29.and.infections.html?f=m1

[10] Tsourdi E Gruber M Rauner M Blankenburg J Ziemssen T Hofbauer LC Graves’ disease after treatment with alemtuzumab for multiple sclerosis. Horm Athens Greece (2015) 14:148–53. 10.14310/horm.2002.1501 25402383

[11] Comi G Freedman MS Kappos L Olsson TP Miller AE Wolinsky JS Pooled safety and tolerability data from four placebo-controlled teriflunomide studies and extensions. Mult Scler Relat Disord (2016) 5:97–104. 10.1016/j.msard.2015.11.006 26856952

[12] Martinelli Boneschi F Vacchi L Rovaris M Capra R Comi G Mitoxantrone for multiple sclerosis. Cochrane Database Syst Rev (2013):CD002127 10.1002/14651858.CD002127.pub3 23728638 PMC11745300

[13] Perini P Calabrese M Rinaldi L Gallo P. The safety profile of cyclophosphamide in multiple sclerosis therapy. Expert Opin Drug Saf (2007) 6:183–90. 10.1517/14740338.6.2.183 17367264

[14] Xing L Dai Z Jabbari A Cerise JE Higgins CA Gong W Alopecia areata is driven by cytotoxic T lymphocytes and is reversed by JAK inhibition. Nat Med (2014) 20:1043–9. 10.1038/nm.3645 25129481 PMC4362521

[15] Gilhar A Etzioni A Paus R Alopecia areata. N Engl J Med (2012) 366:1515–25. 10.1056/NEJMra1103442 22512484

[16] McElwee KJ Freyschmidt-Paul P Hoffmann R Kissling S Hummel S Vitacolonna M Transfer of CD8(+) cells induces localized hair loss whereas CD4(+)/CD25(-) cells promote systemic alopecia areata and CD4(+)/CD25(+) cells blockade disease onset in the C3H/HeJ mouse model. J Invest Dermatol (2005) 124:947–57. 10.1111/j.0022-202X.2005.23692.x 15854035

[17] Bin Saif GA. Severe subtype of alopecia areata is highly associated with thyroid autoimmunity. Saudi Med J (2016) 37:656–61. 10.15537/Smj.2016.6.13777 27279512 PMC4931647

[18] Kasumagić-Halilović E. Thyroid autoimmunity in patients with alopecia areata. Acta Dermatovenerol Croat (2008) 16:123–5. 18812059

[19] Noso S Park C Babaya N Hiromine Y Harada T Ito H Organ specificity in autoimmune diseases: thyroid and islet autoimmunity in alopecia areata. J Clin Endocrinol Metab (2015) 100:1976–83. 10.1210/jc.2014-3985 25734250

[20] Goh C Finkel M Christos PJ Sinha AA Profile of 513 patients with alopecia areata: associations of disease subtypes with atopy, autoimmune disease and positive family history. J Eur Acad Dermatol Venereol (2006) 20:1055–60. 10.1111/j.1468-3083.2006.01676.x 16987257

[21] Petukhova L Duvic M Hordinsky M Norris D Price V Shimomura Y Genome-wide association study in alopecia areata implicates both innate and adaptive immunity. Nature (2010) 466:113–7. 10.1038/nature09114 20596022 PMC2921172

[22] Seyfert S Klapps P Meisel C Fischer T Junghan U. Multiple sclerosis and other immunologic diseases. Acta Neurol Scand (1990) 81:37–42. 10.1111/j.1600-0404.1990.tb00928.x 2330813

[23] Mirzoyev SA Schrum AG Davis MDP Torgerson RR Lifetime incidence risk of alopecia areata estimated at 2.1% by Rochester Epidemiology Project, 1990-2009. J Invest Dermatol (2014) 134:1141–2. 10.1038/jid.2013.464 24202232 PMC3961558

[24] Safavi KH Muller SA Suman VJ Moshell AN Melton LJ 3rd. Incidence of alopecia areata in Olmsted County, Minnesota, 1975 through 1989. Mayo Clin Proc (1995) 70:628–33. 10.1016/S0025-6196(11)63913-X 7791384

[25] Villasante Fricke AC Miteva M Epidemiology and burden of alopecia areata: a systematic review. Clin Cosmet Investig Dermatol (2015) 8:397–403. 10.2147/CCID.S53985 PMC4521674 26244028

[26] Tan E Tay Y-K Goh C-L Chin Giam Y The pattern and profile of alopecia areata in Singapore – a study of 219 Asians. Int J Dermatol (2002) 41:748–53. 10.1046/j.1365-4362.2002.01357.x 12452996

